# Factors affecting prehospital delay in rural and urban patients with stroke: a prospective survey-based study in Southwest Germany

**DOI:** 10.1186/s12883-020-01999-4

**Published:** 2020-12-05

**Authors:** Matthias N. Ungerer, Loraine Busetto, Nima H. Begli, Katharina Riehle, Jens Regula, Christoph Gumbinger

**Affiliations:** Department of Neurology, Heidelberg University Hospital, Im Neuenheimer Feld 400, 69120 Heidelberg, Germany

**Keywords:** Prehospital emergency care, Stroke, Rural communities, Rural health services, Treatment delay

## Abstract

**Background:**

Reducing prehospital delay plays an important role in increasing the thrombolysis rate in patients with stroke. Several studies have identified predictors for presentation ≤4.5 h, but few compared these predictors in urban and rural communities. We aimed to identify predictors of timely presentation to the hospital and identify possible differences between the urban and rural populations.

**Methods:**

From January to June 2017, we conducted a prospective survey of patients with stroke admitted to an urban comprehensive stroke centre (CSC) and a rural primary care centre (PCC). Predictors were identified using binary logistical regression. Predictors and patient characteristics were then compared between the CSC and PCC.

**Results:**

Overall, 459 patients were included in our study. We identified hesitation before seeking help, awareness of the existence of a time-window, type of admission and having talked about stroke symptoms with friends/relatives who had previously had a stroke as the strongest predictors for presentation to the emergency room ≤4.5 h. Patients admitted to the rural PCC were more hesitant to seek help and less likely to contact emergency services, even though patients had comparable knowledge pertaining to stroke care concepts.

**Conclusions:**

Patients from rural areas were more likely to be hesitant to seek help and contacted the EMS less frequently, despite similar self-awareness of having a stroke.

Educational campaigns should focus on addressing these disparities in rural populations. Affected patients should also be encouraged to talk about their symptoms and take part in educational campaigns.

## Background

Thrombolysis shows a time-dependent treatment effect within the therapeutic time-window of 4.5 h in stroke, although new treatment approaches, such as perfusion and MRI guided thrombolysis and thrombectomy, have extended this time-window for treatment in individual cases [1]. Avoidable prehospital delays often contribute to presentations outside of the 4.5-h time-window. Therefore, awareness and recognition of stroke symptoms and efficient prehospital management are critical for the administration of thrombolysis [2].

Patient education, emergency personnel education, advance notification of emergency rooms (ER), triage systems, simplification of validated clinical scales and teleneurology approaches have been successfully implemented to reduce prehospital delays and unnecessary hospital transfers [3]. Lack of general knowledge of stroke is one of the factors responsible for delayed notification of emergency medical services (EMS) and is being addressed in educational campaigns [4]. Different approaches to increase awareness of stroke in the general population, ranging from large scale educational campaigns to online information campaigns are described in the current literature [5]. Patients living in rural communities are more at risk of presentation beyond the time window for thrombolysis [6]. Therefore, novel strategies to reach rural communities, such as telehealth stroke education, have been implemented [7]. Despite these efforts, relevant deficits in knowledge of stroke risk factors and particular stroke symptoms persist and can vary strongly in rural communities [8, 9]. It is critical to understand the reasons for this knowledge gap in stroke care and study other factors that may affect prehospital delays to address these disparities effectively and improve educational campaigns in both developed and developing countries [6, 10].

We conducted a prospective survey-based study of patients admitted with confirmed stroke to a university-level comprehensive stroke centre (CSC) in an urban area and its closely affiliated primary care centre (PCC) in a rural area in southwest Germany to compare the factors affecting prehospital delay between urban and rural communities.

## Methods

### Setting

The CSC is a university hospital and one of the largest healthcare providers with a mostly urban catchment area in southwest Germany. Its neurological department provides the highest standard of stroke care, a specialized neurological ER with access to thrombectomy services 24/7, in a mostly urban area. The PCC is a tertiary teaching hospital in a mostly rural region that is closely affiliated with the CSC. The hospital has a department of neurology staffed during office hours by neurologists trained at the CSC and has access to teleneurology services provided by the CSC during nights and on weekends. The PCC is the primary stroke care provider for its region. Both hospitals have certified stroke units and provide treatment according to current stroke guidelines. Patients that required thrombectomy were transferred to the CSC. This study was approved by the local ethics committee of the university hospital (S306–2016).

### Inclusion and exclusion criteria

Patients admitted with a transient ischaemic attack, stroke or intracerebral hemorrhage as their main hospital diagnoses in a 6 month period were eligible for participation. Patients were recruited consecutively. Exclusion criteria were the inability to provide informed consent, e.g. due to clinical conditions, including aphasia, severe stroke, dementia, language barrier, discharge < 24 h or death.

### Data collection

Consent for the study was obtained at the time of admission. Patients completed a self-questionnaire. A second survey was completed by the treating physician to collect information on comorbidities. Only close-ended questions distributed in the local language (German) were used in this study. A translation of the survey into English can be found in the supplemental materials (Table S1).

### Statistical analysis

Patient characteristics were described using standard descriptive statistics. Admission types were summarized into three categories (self-admission, family physician or transfer from another hospital/nursing home, EMS). Time to presentation was dichotomized into two time-windows (≤4.5 h and > 4.5 h). The primary endpoint of our study was to determine factors that influence timely presentation (≤4.5 h) to the ER (as the PCC did not provide thrombectomy) using binary logistical regression analysis (strength of association was expressed using Nagelkerke’s R squared). We identified the strongest predictors using a stepwise binary logistical regression model and expressed association using odds ratios. The secondary endpoint was to compare these factors in urban and rural populations. The level of significance was set at 0.05 (two-sided) and two-sided 95% confidence intervals (CIs) for all statistical tests. Analyses were conducted with SPSS 22.0 (IBM Corp., Armonk, NY).

## Results

### Baseline characteristics

Overall, 459 patients were included in our study. Three patients were not included due to missing time-to-presentation. The mean age was 69.3 ± 13.2 years. Twenty-two (4.8%), 344 (74.9%) and 93 (20.3%) patients were diagnosed with cerebral hemorrhage, cerebral infarction or transient ischaemic attack, respectively. Of these, 296 (64.5%) and 163 (35.5%) patients were presented in ≤4.5 h and > 4.5 h to the emergency ward. Furthermore, 209 (45.5%) patients were brought in by the emergency services, 94 (20.5%) walked into the ER and 156 (34.0%) patients were sent to the ER by their family physician. The median mRS and NIHSS at presentation were 2 (IQR 1–3) and 2 (IQR 1–5), with the median premorbid mRS as 0 (IQR 0–2). Patient characteristics by dichotomized time-window to presentation are shown in Table 1.

**Table 1:** Baseline characteristics according to time-window at presentation

Characteristic	≤4.5 h	> 4.5 h	All patients
Number of patients, n	296	163	459
Age, mean (SD)	70.47 (12.85)	67.29 (13.57)	69.34 (13.20)
Female, n (%)	137 (46.3)	79 (48.5)	216 (47.1)
Distance to hospital (km), mean (SD)	16.0 (12.7)	17.56 (12.7)	16.56 (12.7)
NIHSS at admission, median (IQR)	3 (1–6)	2 (1–4)	2 (1–5)
pmRS, median (IQR)	1 (0–2)	0 (0–2)	0 (0–2)
mRS at admission, median (IQR)	3 (1–3)	2 (1–3)	2 (1–3)
Type of stroke (ischaemic and TIA), n (%)	288 (97.3)	149 (91.4)	437 (95.2)
Comorbidities			
Arterial hypertension, n (%)	230 (77.7)	115 (70.6)	345 (75.2)
Hyperlipoproteinämie, n (%)	132 (44.6)	63 (38.7)	195 (42.5)
Diabetes mellitus, n (%)	67 (22.6)	26 (16.0)	93 (20.3)
Recurrent Stroke, n (%)	63 (21.3)	32 (19.6)	95 (20.7)
Atrial fibrillation, n (%)	73 (24.7)	24 (14.7)	97 (21.1)
Highest professional degree^a^			
None, n (%)	62 (21.2)	25 (16.0)	87 (19.4)
Apprenticeship, n (%)	174 (59.4)	100 (64.1)	274 (61.0)
University degree, n (%)	57 (19.5)	31 (19.9)	88 (19.6)

*Abbreviations*: *BMI* body mass index, *IQR* interquartile range, *mRS* morbid Rankin Scale, *NIHSS* National Institutes of Stroke Scale, *pmRS* premorbid Rankin scale, *SD* standard deviation, *TIA* Transient ischaemic attack

^a^some missing values

### Predictors for presentation to the ER within a timely time-window of 4.5 h

We identified the following associations of factors with the dichotomized time-window of presentation (crude ratios): hospital of admission was associated with time-window of admission (r squared = 0.02; *p* = 0.008). TIA as diagnosis at discharge was significantly associated with a time-to-presentation ≤4.5 h (r squared 0.053; ICH vs. TIA *p* < 0.001 and stroke vs. TIA *p* = 0.002). Results for a regression analysis for presentation in ≤4.5 h according to diagnosis at discharge can be found in the supplemental materials (Table S2). Patients who recognized stroke symptoms by themselves were more likely to arrive in a timely time-window (r squared = 0.047; *p* < 0.001). Admission type to the ER was significantly associated with timely presentation, with arrival by EMS being most frequently associated with timely presentation (r squared = 0.111; *p* < 0.001). Patients who did not hesitate to seek help were also more likely to present to the ER ≤4.5 h (r squared = 0.211; *p* < 0.001). Having talked about stroke symptoms with friends/relatives who had previously had a stroke and having heard of educational campaigns were both identified as predictors for timely presentation (r squared = 0.073; *p* = 0.001 and r squared = 0.019; *p* = 0.013). Knowledge of potential treatments, in particular concerning the existence of a critical time-window (r squared = 0.023; *p* = 0.007) and of specific treatment regimens (r squared = 0.015; *p* = 0.026) and being aware that help should be sought immediately (r squared = 0.025; *p* = 0.004) were also identified as predictors for a presentation ≤4.5 h. Indicators of socioeconomic status (employment status, education status, household composition) were not identified as predictive factors. Stroke severity at admission, measured by both NIHSS (r squared = 0.047; *p* < 0.001) and mRS (r squared = 0.024; *p* = 0.005), was a non-modifiable factor that predicted early presentation. Distance from hospital in km was not associated with time window of presentation (r squared 0.005; *p* = 0.218).

The strongest predictors were identified using an adjusted stepwise binary regression analysis model: Results of the baseline binary regression model with all predicting factors can be found in Table 2. The following are the results of the optimized stepwise binary regression model (odds for presentation ≤4.5 h): Hesitation before seeking help [0.17 (95% CI 0.08–0.36); *p* < 0.001], awareness of the existence of a time-window [4.62 (95% CI 1.81–11.79); *p* = 0.001], type of admission [self vs EMS: 0.22 (95% CI 0.09–0.56); *p* = 0.002 and family physician and transfer from other hospitals vs EMS: 0.17 (95% CI 0.07–0.41); *p* < 0.001], having talked about stroke symptoms with friends/relatives who had previously had a stroke [2.43 (95% CI 1.15–5.15); *p* = 0.021] and diagnosis at discharge [ICH vs. TIA: 0.08 (95% CI 0.01–0.51); *p* = 0.007 and stroke vs. TIA: 0.35 (95% CI 0.13–0.93); *p* = 0.034] were the strongest predictors for a presentation to the ER in ≤4.5 h.

**Table 2:** Results of binary regression analysis with all predictors (Prediction of time-to-presentation ≤ 4.5 h)

Predictor	***p***-value	Exposure	95% CI	
Hospital of admission (CSC vs PCC)	0.528	1.354	0.529	3.467
Self-observation of stroke symptoms (yes vs no)	0.284	1.550	0.695	3.455
Hesitation to seek help (yes vs no)	**< 0.001**	0.189	0.088	0.407
Talked about stroke symptoms with friends/relatives who had previously had a stroke (yes vs no)	**0.017**	2.650	1.192	5.890
Having heard of educational campaigns (yes vs no)	0.673	1.191	0.529	2.683
Awareness of time window (yes vs no)	**0.003**	4.601	1.668	12.686
Awareness of treatment regimens (yes vs no)	0.930	0.961	0.397	2.329
Knowing to seek help immediately (yes vs no)	0.173	2.280	0.697	7.455
Number of risk factors (1–6)	0.660	1.051	0.842	1.312
NIHSS at admission (1–35)	0.209	1.071	0.962	1.191
Admission type				
self vs EMS	**0.009**	0.253	0.090	0.713
Family physicians and other hospitals vs EMS	**0.02**	0.215	0.082	0.565
Diagnosis at discharge				
ICH vs. TIA	**0.012**	0.083	0.012	0.537
Stroke vs. TIA	**0.023**	0.289	0.099	0.845

*Abbreviations*: *CI* confidence interval, *CSC* comprehensive stroke centre, *EMS* emergency medical services, *ICH* Intracranial hemorrhage, *NIHSS* National Institutes of Stroke Scale, *PCC* primary care centre, *TIA* transient ischemic attack

### Comparison of characteristics and predictors between the urban and rural populations

A total of 343 (74.7%) patients were treated within the urban catchment area of the CSC, while 116 (25.3%) patients were treated at the PCC. Age at presentation was higher in the rural area (*p* = 0.041). Distance (km) from the location at the time of the stroke to the admitting hospital was slightly greater for the CSC (17.3 km vs 14.5 km; *p* = 0.04). The premorbid mRS was similar between both hospitals (*p* = 0.539). Both the mRS and NIHSS at admission were higher at the CSC (*p* = 0.001 and *p* < 0.001). Patients were more likely to present ≤4.5 h at the CSC (*p* = 0.008). Employment status (*p* = 0.777), household composition (*p* = 0.170) and highest professional degree (*p* = 0.149) were similar in both populations.

Here, the strongest predictors for the timely presentation (primary endpoint) between both hospital settings with relevant patient characteristics were compared (Table 3). Patients at the PCC were less frequently presented by emergency services (50.7%, urban; 30.2%, rural) and more likely send to ER via their family physician (29.2%, urban; 48.3%, rural) (*p* < 0.001) and were also more likely hesitant when seeking help (35.3%, urban; 50.5%, rural; *p* = 0.004). Patients in both settings hoped that symptoms would improve spontaneously, although this explanation was more frequent in the population admitted to the CSC (90.8%, urban; 76.0%, rural). Other reasons were fear (8.4 and 14.0%) and limited accessibility (0 and 6%). Interestingly, although patients used EMS less often, the relative number of patients that recognized stroke symptoms themselves (*p* = 0.644) and knowledge of a time-window (*p* = 0.536) for stroke management were similar in both settings. Patients treated in the CSC were more knowledgeable about specific treatment regimens that could be offered (*p* = 0.001).

**Table 3:** Comparison of factors between the comprehensive stroke centre and primary care centre

Characteristic	CSC	PCC	Test used	***p***-value
Number of patients, n	343	116		
Age at admission, mean (SD)	68.61 (13.15)	71.50 (13.10)	t-test	**0.041**
Admission by emergency services, n (%)	174 (50.72)	35 (30.17)	chi-square	**< 0.001**
Hesitated before asking for help, n (%)^a^	121 (35.27)	56 (50.45)	chi-square	**0.004**
Self-observation of stroke symptoms, n (%)^a^	134 (39.30)	48 (41.74)	chi-square	0.644
Talked about stroke with friends/relatives who had previously had a stroke, n (%)^a^	79 (47.02)	27 (55.10)	chi-square	0.320
Knowledge of stroke management				
Awareness of time-window, n (%)	65 (18.95)	19 (16.38)	chi-square	0.536
Awareness of treatment regimens, n (%)	90 (26.24)	13 (11.21)	chi-square	**0.001**
Number of risk factors, median (IQR)	2 (1–4)	2 (1–3)	Mann–Whitney-U-test	0.816
NIHSS at admission, median (IQR)	3 (1–6)	1 (0–3)	Mann–Whitney-U-test	**< 0.001**
mRS at admission, median (IQR)	2 (1–3)	2 (1–3)	Mann–Whitney-U-test	**0.001**

*Abbreviations*: *BMI* body mass index, *CSC* comprehensive stroke centre, *IQR* interquartile range, *mRS* morbid Rankin Scale, *NIHSS* National Institutes of Stroke Scale, *PCC* primary stroke centre, *SD* standard deviation

^a^some missing values

## Discussion

### Main findings

We found that awareness of the existence of a time-window, type of admission, having talked about stroke symptoms with friends/relatives who had previously had a stroke and seeking help without hesitation strongly predicted timely presentation (≤4.5 h) of stroke patients to the ER. We identified several additional factors that were associated with time-window to presentation. However, social status was not a predictor and both rural and urban populations had a similar understanding of the basic principles of stroke, such as awareness of a critical time window for the treatment of stroke and the ability of patients to recognize they were having a stroke. The main difference in the predictors of timely presentation between the CSC and PCC was that the rural population contacted the EMS less frequently and more often hesitated to seek help, even though both groups had a comparable knowledge of stroke concepts and a similar ability to recognize they were having a stroke.

### Comparison with previous studies

Several factors we identified as predictors for the timely presentation contributed to prehospital delay in other studies: stroke severity, recognition of stroke symptoms and immediate contact to the EMS have been shown to reduce prehospital delays in published literature, similar to our study [11, 12]. One of the strongest predictors for a delayed presentation was the active hesitation of patients in seeking help, believing that the symptoms would resolve on their own. This has been described in previous publications, although the reasons for this phenomenon remain unclear [13, 14]. Our analysis contributes to previous findings by comparing these relevant predictors between rural and urban populations in a standardized manner.

Differences in socioeconomic status have been used to explain why patients react to stroke symptoms differently and tend to hesitate before seeking medical attention [15]. In our study population, socioeconomic status was not a predicting factor and differences in social status between the rural and urban populations were insignificant, suggesting a homogenous distribution of socioeconomic status in both areas.

Several studies have described deficits in stroke knowledge in rural populations in addition to sociodemographic factors to account for differences in behaviour [10, 16]. We observed that the response to stroke symptoms was different despite similar knowledge of basic stroke principles (critical time window, the awareness that patients have a stroke). There were small differences in the knowledge of specific stroke symptoms and treatment options, which insufficiently accounted for the differences in rates of timely presentation. The reasons for the different reactions to stroke symptoms despite similar knowledge of stroke in patients from rural communities needs further research. This disparity should also be addressed in future educational campaigns.

### Implications of findings

Our findings evidently suggest that educational campaigns should increase the awareness of critical time-window and encourage stroke survivors to talk about their symptoms and general experience. We also showed that rural populations particularly were more hesitant in seeking help and contacted the EMS less often. Future studies should further explore the reasons for patient hesitation as these should be addressed in educational campaigns. Educational campaigns should also focus on increasing the awareness of these issues in rural areas and address the disparities in reaction to stroke symptoms to improve the time-window to presentation in rural areas.

### Strengths and limitations

The main strengths of this study are the completeness of data and prospective study design. Imputation of data was not necessary. We studied the results of a large patient cohort from two distinct catchment areas that allowed a direct comparison of an urban and rural population using the same survey. The second part of the survey was completed by the treating physician, ensuring completeness and accuracy of clinical data, such as stroke severity and comorbidities.

The main limitation is that the randomization of patients was not possible and assessment of patient knowledge was limited by the number and nature of close-ended questions asked. There is an inherent risk for a recall bias in this type of study. Also, the survey was not previously validated. Importantly, patients admitted to the CSC were more severely affected, suggesting that stroke severity could be a potential confounder. However, stepwise binary regression analysis demonstrated that when other predicting factors were adjusted, both hospital admission and stroke severity no longer were significant predictors of the time-window to presentation and were, therefore, not included in the optimized regression model, making a significant bias by stroke severity or hospital properties unlikely. Mean distance to hospital was also slightly greater in the urban cohort, although this factor was not identified as a predictor. It is still possible that both accessibility to medical services as well as differences in access to public health education contributed to disparities in timely presentation and certain aspects of knowledge on stroke (i.e. specific treatment possibilities) in the rural population. However we found that the number of patients that realized that they were having a stroke themselves and had knowledge of a critical time window, both important motivators for a timely presentation, were similar in-between both populations. The median NIHSS and mRS at admission were also low, reflecting the fact that many severely affected patients were unable to complete the questionnaire and were, therefore, not eligible to take part in this study. This may lead to selection bias and limits the generalizability of our results to more severely affected patients. This could have been addressed by allowing for relatives/surrogates to respond in place of the patient, however this was not envisaged in the study design. Other vulnerable patient groups, such as patients with dementia who made up only 2% of our patient population, are also underrepresented for the same reasons. Further studies are necessary to investigate factors influencing time to presentation in these patient subgroups.

### Conclusion

We observed that hesitation to seek help, awareness of critical time-window, talking about stroke symptoms with friends/relatives who had previously had a stroke and admission type were the strongest predictors for early presentation to the ER. Importantly, patients from rural areas were more likely to be hesitant to seek help and contacted the EMS less frequently, despite similar self-awareness of having a stroke.

Future efforts to reduce prehospital delays in rural populations should not only focus on recognition of stroke symptoms but also on reducing patient hesitation and encourage patients to contact the EMS immediately, irrespective of the duration of stroke symptoms, to increase the rate of patients arriving in an early time-window for stroke management. Affected patients should also be encouraged to talk about their symptoms and take part in educational campaigns.

## Supplementary Information

**Additional file 1: Table S1.** Survey.

**Additional file 2: Table S2.** Bivariate Regression analysis for presentation in ≤4.5h according to diagnosis at discharge.

## Data Availability

Data and code can be made available on request.
