# Supplementary material for: Factors affecting prehospital delay in rural and urban patients with stroke: a prospective survey-based study in Southwest Germany
Source: BMC Neurol. 2020 Dec 5;20:441. doi: 10.1186/s12883-020-01999-4 (PMC7718652; doi:10.1186/s12883-020-01999-4)
Supplement: Supplementary file 1 — Additional file 1: Table S1. Survey. [file 12883_2020_1999_MOESM1_ESM.pdf]

Date of survey:
Abbreviation (physician):

Pat-ID:

Study on awareness of and reaction to stroke symptoms

Survey

Awareness of stroke symptoms

□ Self-awareness of symptoms

□ Symptoms were first recognized by someone else

Time of stroke

□ wake-up / unknown

□ Date and time of first symptoms: __________ & __________

Did you interpret your symptoms to be indicative of a stroke?

□ Yes □ No

How did you reach the hospital?

□ by myself

□ Family physician

□ by phone

□ direct contact

What recommendation did your family physician give you?

□ Hospitalization by emergency medical services

□ Self-presentation at the nearest hospital

Time from symptom onset to presentation to family physician (in minutes): _________

□ Hospitalization initiated by mobile nursing service

□ Hospitalization by emergency medical services

□ Hospitalization initiated by medical on-call service

□ Transfer from another hospital

What symptoms did you have [Multiple symptoms possible]?

□ Half-sided weakness □ Half-sided paresthesia

□ Slurred speech □ Word finding disorder

□ Vision impairment

□ Double vision □ Drowsiness / Dizziness

□ Vomiting / Nausea □ Headache

□ Other: ______________________________________

Duration of symptoms?

□ Symptoms disappeared before presentation at the emergency room

□ Symptoms persisted at the time of presentation

Did you hesitate before you contacted emergency services?

□ No □ Yes Reasons: □ Accessibility □ Fear □ hope that symptoms would resolve spontaneously

□ other

Has a family member / friend / neighbor of yours suffered a stroke?

□ Yes □ No

If yes: Did you discuss the stroke symptoms with this person?

□ Yes □ No

Did you obtain knowledge on stroke from educational campaigns?

□ No □ Yes, if yes, what was the name of the educational campaign? ______________

Have you heard of educational campaigns (i.e. “BaWü gegen den Schlaganfall”)?

□ No □ Yes

Have educational campaigns influenced your reaction to stroke symptoms?

□ No □ Yes

Which symptoms would you associate with a stroke [Multiple answers possible!]?

□ Half-sided weakness □ Half-sided paresthesia

□ General weakness □ Slurred speech

□ Word finding disorder □ Fever

□ Vision impairment □ Double vision

□ Headache □ Dizziness / Drowsiness

□ Vomiting / Nausea

Are you familiar with treatments administered in acute stroke?

□ No □ Yes If yes, which ones?_____________

Are you aware of a time-window for treatment of acute stroke?

□ No □ Yes If yes, how many hours/minutes?_____________

Did you know that you should have called medical professionals immediately (i.e. 19222, 112,…)?

□ No □ Yes

Time to arrival at hospital after first symptoms: _______min

With everything you know now: Did you react correctly to your first stroke symptoms?

□ No □ Yes

Did you require assistance at home before the stroke?

□ None

□ some help (i.e. help with shopping)

□ regular help (i.e. Mobile nursing care)

□ constant nursing care / nursing home

Housing

□ alone □ with spouse □ with other relatives

□ in a nursing home □ Other__________________

Employment

□ Pensioner □ unemployed □ employed □ taking care of the household

School-leaving qualifications

□ None □ “Hauptschule” □ “Realschule” □ “Gymnasium” □ Other:__________

Professional qualifications

□ None □ Apprenticeship □ University degree

We thank you for taking part in this survey!

To be completed by the treating physician!

Calculated Body Mass Index: ______kg/m^2

Preexisting medical conditions

Arterial hypertension	□ Yes	□ No
Hypercholesterinemia	□ Yes	□ No
Diabetes mellitus	□ Yes	□ No
Previous stroke	□ Yes	□ No
Atrial fibrillation	□ Yes	□ No
Cardiac pacemaker	□ Yes	□ No
Dementia	□ Yes	□ No
Coronary artery disease	□ Yes	□ No
Excessive alcohol consumption	□ Yes	□ No
Smoking	□ Yes	□ No
Peripheral vascular disease	□ Yes	□ No

Distance to the hospital: ______km

Height: ______ cm Weight: ______kg

Calculated Body Mass Index:______kg/m^2

NIHSS at admission:____

Premorbid Rankin Score (0-5):____

mRS at admission (0-5):____

Imaging modality □ CCT □ CMRT

Stroke in imaging □ ja □ nein

Type of stroke □ Hemorrhagic stroke □ Ischemic stroke □ TIA

Discharge diagnosis as ICD:

Discharge to

□ home □ Rehabilitation facility □ other hospital □ Nursing home

If transfer to another hospital: why? □ Treatment of pneumonia □ PEG tube

□ cardiac pacemaker □ Early rehabilitation □ Wait until transfer to rehabilitation facility

□ insufficient nursing care at home □ Other: _______
