# Supplementary material for: Factors affecting prehospital delay in rural and urban patients with stroke: a prospective survey-based study in Southwest Germany
Source: BMC Neurol. 2020 Dec 5;20:441. doi: 10.1186/s12883-020-01999-4 (PMC7718652; doi:10.1186/s12883-020-01999-4)
Supplement: Supplementary file 2 — Additional file 2: Table S2. Bivariate Regression analysis for presentation in ≤4.5h according to diagnosis at discharge. [file 12883_2020_1999_MOESM2_ESM.docx]

| Variable | ICH | | Ischemia | | TIA | |
| --- | --- | --- | --- | --- | --- | --- |
|  | r squared | p-value | r squared | p-value | r squared | p-value |
| Hospital | (*) | (*) | 0.061 | **<0.001** | 0.001 | 0.817 |
| Self-recognition | 0.080 | 0.267 | 0.024 | **0.016** | 0.103 | **0.018** |
| Admission type | 0.004 | 0.970 | 0.134 | **<0.001** | 0.152(**) | **0.001(**)** |
| Hesitation to seek help | 0.00 | 0.933 | 0.240 | **<0.001** | 0.244 | **<0.001** |
| Having talked about stroke symptoms with friends/relatives who had previously had a stroke | (*) | (*) | 0.046 | **0.022** | 0.046 | 0.238 |
| Having heard of educational campaigns | 0.061 | 0.320 | 0.010 | 0.121 | 0.046 | 0.106 |
| Knowledge of critical time window | (*) | (*) | 0.017 | **0.042** | 0.011 | 0.431 |
| Knowledge of specific stroke treatments | 0.018 | 0.605 | 0.021 | **0.026** | 0.005 | 0.597 |
| Awareness that help should be sought immediately | 0.044 | 0.407 | 0.014 | 0.059 | 0.061 | **0.048** |
| NIHSS at admission | 0.076 | 0.291 | 0.100 | **<0.001** | 0.032 | 0.237 |
| Number of risk  factors | 0.015 | 0.629 | 0.016 | **0.046** | 0.025 | 0.235 |

Table S2: Bivariate Regression analysis for presentation in ≤4.5h according to diagnosis at discharge.

(*) insufficient data ; (** ) Chi-squared test
